# Utilization of telemedicine to improve burn care in a developing country

**DOI:** 10.1186/cc12464

**Published:** 2013-03-19

**Authors:** J Knittel, G Fuzaylov

**Affiliations:** 1 Massachusetts General Hospital, Boston, MA, USA

## Introduction

Our objective is to present our experience from Shriner's Hospital and Massachusetts General Hospital in Boston, MA, USA in using telemedicine to provide acute burn and critical care consultation on pediatric and adult burn patients in Lviv, Ukraine, as well as in triage and transport of critically ill patients from Lviv to a tertiary-care facility in the USA for further management.

## Methods

Using a new telemedicine learning center established at City Hospital #8 in Lviv, Ukraine, consultations regarding acutely injured burn victims occurred between physicians in Ukraine and physicians at Shriners Hospital and Massachusetts General Hospital in Boston. After the initial presentation, each patient was reviewed on a daily basis by physicians in Boston. Skype, an Internet-based communication tool, was used in communication with the Burn Center in Lviv. Radiographic images were scanned and digitalized using an electronic scanner, and JPEG image compression was used to facilitate the transmission of radiographic images and patient charts. Informed consent and HIPPA guidelines were followed in transmitting any patient-related information.

## Results

Since 2011 we have provided consultation on 14 patients in Lviv, Ukraine, ranging in age from 15 months to 63 years. Each patient had an average of six consultations. We present two of these cases as examples of the capabilities of our telemedicine program. The first case involved a 15-month-old female with 40% TBSA from scald injury, where telemedicine was instrumental in the primary assessment as well as to arrange a direct assessment from a nearby burn surgeon. The second case resulted from a house fire with multiple casualties, where physicians in Boston were able to utilize telemedicine to guide the initial resuscitation and airway management of three critically burned children, as well as to arrange for transport of one of the victims, an 11-year-old male with 87% TBSA, from Ukraine to the USA for acute management. Multiple difficulties were overcome in implementing the system between the two countries including: time zone differences, language barrier, and different approaches to patient care.

## Conclusion

We have established a telemedicine program linking physicians in Boston, MA, USA with City Hospital #8 in Lviv, Ukraine to improve care in pediatric and adult burn patients. Our program has provided consultation on 14 patients since 2011, and it highlights the capabilities of telemedicine for acute consultation as well as triage and transport of critically ill patients to tertiary-care facilities.

